# Rationale, design and methodology of the image analysis protocol for studies of patients with cerebral small vessel disease and mild stroke

**DOI:** 10.1002/brb3.415

**Published:** 2015-11-26

**Authors:** Maria del C. Valdés Hernández, Paul A. Armitage, Michael J. Thrippleton, Francesca Chappell, Elaine Sandeman, Susana Muñoz Maniega, Kirsten Shuler, Joanna M. Wardlaw

**Affiliations:** ^1^ Department of Neuroimaging SciencesCentre for Clinical Brain SciencesUniversity of EdinburghEdinburghUK; ^2^ Department of Cardiovascular SciencesUniversity of SheffieldSheffieldUK

**Keywords:** Blood–brain barrier, image analysis, MRI, protocol, small vessel disease, stroke

## Abstract

**Rationale:**

Cerebral small vessel disease (SVD) is common in ageing and patients with dementia and stroke. Its manifestations on magnetic resonance imaging (MRI) include white matter hyperintensities, lacunes, microbleeds, perivascular spaces, small subcortical infarcts, and brain atrophy. Many studies focus only on one of these manifestations. A protocol for the differential assessment of all these features is, therefore, needed.

**Aims:**

To identify ways of quantifying imaging markers in research of patients with SVD and operationalize the recommendations from the STandards for ReportIng Vascular changes on nEuroimaging guidelines. Here, we report the rationale, design, and methodology of a brain image analysis protocol based on our experience from observational longitudinal studies of patients with nondisabling stroke.

**Design:**

The MRI analysis protocol is designed to provide quantitative and qualitative measures of disease evolution including: acute and old stroke lesions, lacunes, tissue loss due to stroke, perivascular spaces, microbleeds, macrohemorrhages, iron deposition in basal ganglia, substantia nigra and brain stem, brain atrophy, and white matter hyperintensities, with the latter separated into intense and less intense. Quantitative measures of tissue integrity such as diffusion fractional anisotropy, mean diffusivity, and the longitudinal relaxation time are assessed in regions of interest manually placed in anatomically and functionally relevant locations, and in others derived from feature extraction pipelines and tissue segmentation methods. Morphological changes that relate to cognitive deficits after stroke, analyzed through shape models of subcortical structures, complete the multiparametric image analysis protocol.

**Outcomes:**

Final outcomes include guidance for identifying ways to minimize bias and confounds in the assessment of SVD and stroke imaging biomarkers. It is intended that this information will inform the design of studies to examine the underlying pathophysiology of SVD and stroke, and to provide reliable, quantitative outcomes in trials of new therapies and preventative strategies.

## Introduction

Stroke remains the commonest cause of disability in adulthood and uses at least 5% of the health care budget. Approximately, 30% of sufferers are left disabled (Bamford et al. 1991). At least, 25% of all ischemic strokes are lacunar. Lacunar infarcts (Wardlaw et al. 2013a) coexist with white matter hyperintensities (WMH) (Pantoni 2002; Roman et al. 2002) enlarged perivascular spaces, lacunes, and microhemorrhages that occur silently and are visible on fluid attenuation inversion recovery (FLAIR), T2‐weighted (T2W), T2*‐weighted (T2*W), and T1‐weighted (T1W) magnetic resonance imaging (MRI). These features are part of the same diffuse small vessel disease (SVD) spectrum (Wardlaw et al. 2013a,b). Some of them, for example, microbleeds (Martinez‐Ramirez et al. 2014), vessel mineralization (Glatz et al. 2013), lacunes (Warren et al. 2015), and perivascular spaces (Kwee and Kwee 2007), are visible to the eye, but difficult to detect with current computational image processing methods. Others, like subtle WMH are hard to appreciate visually on radiological images or even histology (Wardlaw et al. 2015). The difficulty in quantifying these features may be a cause of their neglect.

The interpretation and analysis of MRI is complex, and stroke‐ and ageing‐related tissue changes pose complex problems for automated processing algorithms (e.g., poor gray/white matter contrast negatively impacts on reliability of tissue classification). Efforts to identify and overcome these and other issues (e.g., lesion intensity and shape distortion while applying conventional image processing pipelines) that current brain image analysis methods (Wardlaw et al. 2013b) do not yet cope with, are ongoing. Powerful computational image processing tools for analyzing normal adult brains are freely available and widely used (Zhang et al. 2001; Jenkinson et al. 2002; Tustison et al. 2010; Patenaude et al. 2011), but some have limitations when applied to brains with abnormalities, making the development of new tools and image processing protocols to study disease a priority. Therefore, an imaging protocol that allows exploration of the disease manifestations together in all their extent and carefully differentiates features with similar radiological appearance is an urgent need.

Each MRI modality has unique advantages. Diffusion‐weighted MRI (DWI) and diffusion tensor MRI (DT‐MRI) provide unique information on the viability of brain tissue and are particularly sensitive for the detection of acute ischemic stroke, differentiating it from other processes that also cause neurologic deficit (Wardlaw et al. 1997). Parametric maps extracted from diffusion tensor images such as fractional anisotropy (FA) and mean diffusivity (MD) can demonstrate in vivo the alterations in axonal microstructure or interstitial fluid content of visible lesions and the surrounding normal‐appearing tissue (O'Sullivan et al. 2002; Topakian et al. 2010; Uh et al. 2010; Leritz et al. 2014; Muñoz Maniega et al. 2015). The longitudinal relaxation time (T1) provides quantitative information related to brain water content, and is therefore a potential marker for edematous brain tissue (Bastin et al. 2002). Differences in these imaging biomarkers can help identify pathophysiological changes in vivo. On the other hand, on structural MRI, different pulse sequence weightings cause image contrast between tissues to facilitate the assessment of specific SVD markers. For example, T1W images are useful for assessing subcortical gray matter and the cerebral cortex, T2W images are useful for detecting white matter changes and perivascular spaces, and a gradient‐recalled echo T2*W images can facilitate the identification of microbleeds and improve the rate of small lesion detection. In addition, the T2* value can indirectly reflect changes in tissue biochemical components (e.g., change in oxygen saturation, hemorrhages, calcifications, and iron depositions) (Chavhan et al. 2009).

In this work, we combine validated computational image processing pipelines developed in‐house with traditional visual assessment tools in a novel multispectral and multiparametric framework to quantitatively assess all MRI correlates and manifestations of SVD. By combining single processes in a unique workflow, we strive to optimize machine processing time and human interaction, improve user experience, and explore the disease manifestations all together. The scientific purpose is ultimately to determine the core mechanisms of small vessel stroke and other forms of brain damage resulting from small vessel disease. Reliable comprehensive quantification of all disease features is an essential component of achieving that goal. The suitability of this protocol for studying stroke and all other presentations of cerebral SVD is evaluated using imaging data from patients recruited for two studies of stroke mechanisms. Thus, we present a robust image processing protocol, which operationalizes the recommendations of the STandards for ReportIng Vascular changes on nEuroimaging (STRIVE) guidelines (Wardlaw et al. 2013b).

## Methods

### Subjects and primary definitions

#### Subjects

Our protocol was developed and evaluated using data from 250 consecutive patients who presented to a hospital stroke service with the first clinically evident nondisabling (i.e., mRS <3) lacunar or mild cortical ischemic stroke. Diabetes, hypertension, and other vascular risk factors were not criteria for exclusion. However, patients with unstable hypertension or diabetes, other neurological disorders, major medical conditions including renal failure, contraindications to MRI, unable to give consent, those who had hemorrhagic stroke, or those whose symptoms resolved within 24 h (i.e., transient ischemic attack) were excluded. The studies that provided data for evaluating this protocol were approved by the Lothian Ethics of Medical Research Committee (REC 09/81101/54) and the NHS Lothian R+D Office (2009/W/NEU/14), and conducted according to the principles expressed in the Declaration of Helsinki. All patients gave written informed consent. The mean age at baseline was 66‐years old (SD 10 years).

#### Role of imaging in the identification of the stroke subtype

All patients were examined by a stroke physician. The final diagnosis of stroke was determined by an expert panel of stroke physicians, neurologists, and neuroradiologists who considered all available information and decided if the diagnosis was stroke or not, and if stroke, then whether lacunar or cortical subtype according to the Oxfordshire Community Stroke Project classification (Bamford et al. 1991). The clinical diagnosis was modified based on imaging information to assign a final stroke type, thus avoid misclassifying 20% of lacunar/cortical stroke with clinical‐only diagnosis according to a previous estimate (Potter et al. 2010). DWI, fluid attenuation inversion recovery (FLAIR), T2‐, T1‐, and T2*‐weighted MRI were used to identify the acute stroke lesion (Wardlaw et al. 1997; Keir et al. 2004). After examination, the median National Institutes of Health Stroke Scale of the sample was 2 (IQR 1.3).

### Brain MRI acquisition protocol

All MRI scans were acquired using a 1.5T GE Signa Horizon HDxt clinical scanner (General Electric, Milwaukee, WI, USA) operating in research mode and using a self‐shielding gradient set with maximum gradient of 33 mT/m and an 8‐channel phased‐array head coil. Imaging data were obtained soon after presenting to hospital with acute stroke symptoms (median 12 days, IQR 4–27 days) and 1 year (*n* = 200) (Heye et al. 2015) or 3 years (*n* = 50) (Wardlaw et al. 2009) later to provide information on disease progression.

**Figure 1:**
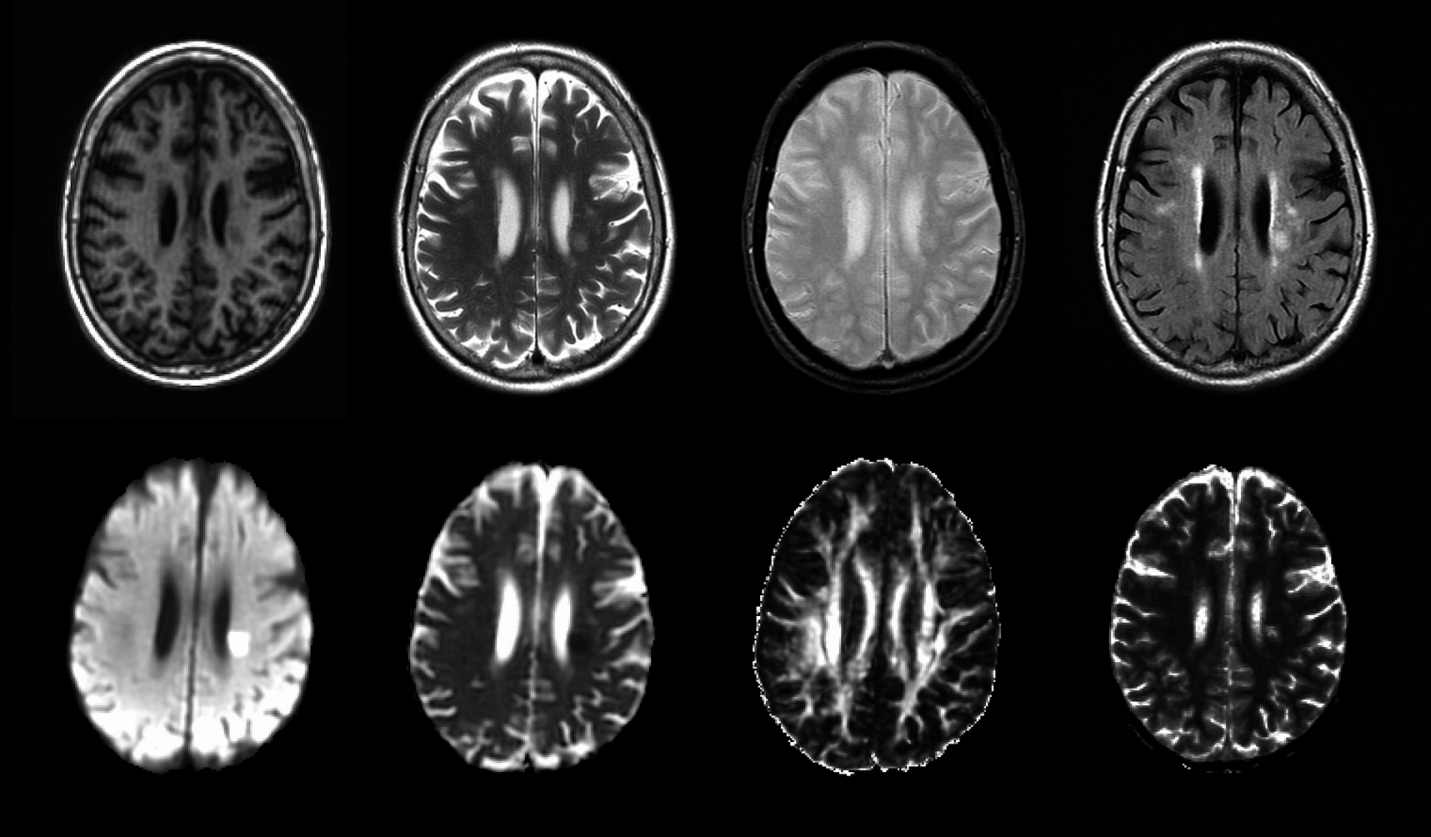
Axial slice from a patient showing the imaging modalities used in the multiparametric approach that this protocol describes. In the upper row, from left to right: structural T1‐weighted (for analyses of volumes and shapes), T2‐weighted (brain tissues, lesions, and perivascular spaces), gradient echo (ICV, brain tissues, lesions, mineral deposition, and microbleeds/hemorrhages) and FLAIR (brain tissues and lesions, especially WMH). In the bottom row, from left to right: diffusion‐weighted image (for identification of stroke lesions) and parametric maps of mean diffusivity, fractional anisotropy and longitudinal relaxation time (T1) (for quantitative tissue integrity analyses).

The acquisition protocol (Table 1) was designed to provide high‐resolution images with multiple contrasts and quantitative parameter maps within a total acquisition time acceptable to mild stroke patients (total acquisition time of approximately 30 min). T2‐weighted (T2W), T2‐weighted fluid‐attenuated inversion recovery (FLAIR), gradient‐recalled echo (GRE), and diffusion tensor imaging (DTI) were acquired axially using two‐dimensional multislice sequences with 5‐mm slice thickness in order to optimize in‐plane resolution and signal‐to‐noise ratio for manual and semiautomatic image processing (Fig. 1). To reduce errors and distortions introduced during image registration, these sequences were acquired with the same spatial coverage and axial orientation (aligned with the midline and the line joining anterior‐ and posterior commissures). To provide a reference image with minimal motion artifact for coregistration, and to enable segmentation of perivascular spaces and other features, the T2W image was acquired using a radial *k*‐space sampling scheme (“PROPELLER”) (Pipe 1999); T2‐FLAIR was obtained for segmentation of hyperintensities. For identification of microbleeds and efficient generation of intracranial masks, a rewound gradient‐recalled echo sequence yielding relatively uniform intensity in brain tissue and CSF was chosen. DTI was acquired for assessment of recent stroke lesions and generation of fractional anisotropy and mean diffusivity maps (Fig. 1). In addition, a three‐dimensional inversion recovery prepared spoiled gradient echo T1W image with thinner slices was acquired to facilitate semiautomated segmentation of subcortical gray matter structures. Finally, proton density‐weighted (*α *= 2°) and T1‐weighted (*α *= 12°) three‐dimensional fast spoiled gradient‐recalled echo (FSPGR) images were acquired to facilitate quantitative T1 mapping; these were obtained as part of a separate visit, but can be acquired in <4 min as part of the combined imaging protocol.

**Table 1:** Imaging protocol from the study used for developing this image analysis protocol, acquired using a1.5T scanner with an 8‐channel head coil

Sequence	DWI/DTI (30 diffusion directions)	FLAIR (TI = 2200 ms)	T2W Fast spin echo	T2*W gradient recalled echo (FA = 20°)	3D T1W (TI = 500 ms, FA = 8°)	FSPGR (FA = 2,12°)
Orientation	Axial	Axial ACPC	Axial ACPC	Axial ACPC	Sagittal	Axial ACPC
TE (ms)	82	153	90	15	2.9	3.1
TR (ms)	7700	9000	6000	800	7.3	8.2
FOV (cm)	24 × 24	24 × 24	24 × 24	24 (AP) × 18	330 (SI) × 214.5	24 × 24
Slice thickness (mm)	5.0	5.0	5.0	5.0	1.8	4
Slice gap (mm)	1.0	1.0	1.0	1.0	0	0
Matrix	128 × 128	384 (AP) × 224	384 × 384	384 (AP) × 168	256 (SI) × 146	256 (AP) × 192
No. slices	28	28	28	28	100	42
Acquisition time	4:14	4:48	2:30	4:32	4:17	1.13

ACPC, Anterior Commissure – Posterior Commissure; W, weighted; FSPGR, fast spoiled gradient echo; FLAIR, fluid attenuation inversion recovery; DTI, diffusion tensor imaging.

A similar protocol could be used at 3T, but we recommend utilizing the increased signal obtained at higher field strength to reduce the slice thickness to 3–4 mm (ideally without slice spacing) for the two‐dimensional acquisitions; 3D T1‐weighted scans may be acquired with 1‐mm isotropic resolution without significant increase in the acquisition time or loss of signal. It should be noted that due to the greater influence of magnetic susceptibility variation at 3T, a shorter echo time should be used for the gradient echo sequence in order to obtain a similar T2* weighting and to reduce artifacts. Finally, standing wave effects, which are also more pronounced at 3T, may reduce the accuracy of T1 when measured using the variable flip angle method; we therefore recommend that a *B*_1_ mapping sequence, or a *B*_1_‐insensitive T1 measurement technique is used (Deoni 2007); these methods will also reduce T1 measurement error at 1.5T due to *B*_1_ variation and missetting of the flip angle.

### Imaging data storage and processing

Imaging data are anonymized and stored on secure backed‐up servers. Unique study identification numbers identify case records. Identifiable information is held in a separate password‐protected database. The quality of all imaging data is visually verified, and the acquisition parameters and relevant imaging information are stored on an imaging database. Fourier‐reconstructed images in DICOM (Digital Imaging and Communications in Medicine standard) format are then viewed in workstations with high‐resolution displays for visual analysis by expert neuroradiologists and converted to Analyze 7.5 and nifti 1.1 formats for further computational analysis. All results from the computational analyses are double checked, independently, and manually edited by trained analysts, supervised by neuroradiologists. Table 3 shows the approximate time that each manual verification/editing step takes for operators with different levels of experience, to guarantee the quality of the research output.

### Structural MRI analysis

We use a combined multiparametric approach of qualitative visual rating scales performed by an expert neuroradiologist and computational image processing methods, both performed blind to all clinical and personal data. These approaches are complementary.

#### Visual ratings

We combine different visual rating scales, all validated, to record the location (Wardlaw and Sellar 1994; The IST‐3 Collaborative Group, 2015), type and size of all cortical and subcortical infarcts (new and old), presence of cortical siderosis, microbleeds, iron deposition, perivascular spaces, WMH, and atrophy (see scores on Table 2) at baseline, as well as their progression at follow‐up. Examples of proformas to record these data can be accessed from http://www.ed.ac.uk/schools-departments/clinical-sciences/neuroimaging-sciences/imaging-services/image-analysis-tools.

**Table 2:** Definitions, visual and computational assessment methods of the normal and pathological imaging features

Imaging feature/parameter	Definition	Assessment method
Intracranial volume (ICV)	Contents inside the inner skull table including brain tissue, cerebrospinal fluid, veins and dura. The inferior limit is considered on the axial slice just superior to the tip of the odontoid peg at the foramen magnum	Automatic delineation on T2*W using the Object Extraction Tool on Analyze^™^, followed by manual correction using the Region of Interest Tool of the same software
Cerebrospinal fluid volume (CSF)	Volume occupied by the cerebrospinal fluid, obtained after removing that occupied by veins, sinuses and dura from the non‐brain tissue region within the ICV	Automatically determined from the voxel‐wise subtraction between non‐brain tissue (this obtained from the fusion of T2*W and FLAIR images) and regions with liquid content (obtained from the fusion of T1W and T2W) using MCMxxxVI (Valdes Hernandez et al. 2012a)
Brain tissue volume	Volume of all nonliquid contents of the intracranial cavity with the exception of the veins, sinuses and dura	*Volume*: ICV minus the volume of CSF, veins, sinuses and dura (i.e., non‐brain tissue). *Scores*: Deep and superficial atrophy rated following a normative age template (Farrell et al. 2009) (see text)
White matter hyperintensities (WMH)	Punctate or diffuse areas in the white matter and deep gray matter of the cerebral hemispheres or in the brainstem that were 3 mm or larger in diameter, and hyperintense with respect to normal‐appearing tissues on T2W and FLAIR. Some hypointensity on T1W is allowed as long as this is not as hypointense as CSF. Hyperintensities subtler than the very obvious ones are included when they have outstanding intensity differences with respect to the white matter considered “normal” as per identified on T1W images	*Properties*: Measured on the FA, MD and T1 maps after multiplying these parametric images by the WMH binary masks. *Volume*: Quantified semiautomatically from the fusion of T2*W and FLAIR using MCMxxxVI (Hernandez et al. 2010) *Scores*: Fazekas visual rating scale (Fazekas et al. 1988): Periventricular hyperintensities scored as: 0 = absence, 1 = “caps” or pencil‐thin lining, 2 = smooth “halo”, and 3 = irregular periventricular hyperintensities extending into the deep white matter. Deep WMH are scored as: 0 = absence, 1 = punctate foci, 2 = beginning confluence of foci, and 3 = large confluent areas. Wahlund visual rating scores (Wahlund et al. 2001): range from 0 (none) to 3 (severe) separately rated in frontal, parieto‐occipital,temporal, infratentorial and basal ganglia regions
Less‐intense white matter hyperintensities	Pale subregions of the WMH corresponding with FLAIR intensity levels that are 5–6 standard deviations above the mean of the normal brain parenchyma sampled on slices across the whole volume, with ill‐defined and diffuse borders. Appear isointense with respect to the normal‐appearing white matter in the T2W sequence and slightly hypointense with respect to the normal‐appearing white matter but undistinguishable in the deep gray matter in T1W	*Properties*: Measured on the FA, MD and T1 maps after multiplying these parametric images by the correspondent binary masks *Volume*: Quantified from binary masks obtained from the logic “and” operation between WMH and WM masks, both resultant from the color fusion of paired structural images using MCMxxxVI (Fig. 2)
Intense (i.e., severe) white matter hyperintensities	Subregion of the WMH with high signal strength on FLAIR, equal to or greater than 50% of the mean of the less‐intense white matter hyperintensities. They also appear hyperintense with respect to the normal‐appearing white matter on T2W and very hypointense on T1W sequences	*Properties*: Measured on the FA, MD and T1 maps after multiplying these parametric images by the correspondent binary masks *Volume*: Quantified from binary masks obtained from the subtraction of the WMH mask from the WM masks, both resultant from the color fusion of paired structural images using MCMxxxVI (Fig. 2)
Perivascular spaces	Cavities that surround small penetrating cerebral arterioles as they course from the subarachnoid space through the brain parenchyma (Kwee and Kwee 2007). They conform to the path of the associated arterial branches and typically present bilaterally. On a transverse/axial T2W MRI, they can be round, oval or curvilinear hyperintensities, linear if running longitudinally in the centrum semiovale or short linear structures at the insular or temporal white matter (Valdes Hernandez et al. 2013)	*Scores*: Potter scale (Potter et al. 2015): rated in the midbrain, basal ganglia, hippocampi and centrum semiovale: 0 = no EPVS, 1 = <10 EPVS, 2 = 10–20 EPVS, 3 = 21–40 EPVS, and 4 > =40 EPVS
Index stroke lesions	At baseline, this includes the region on FLAIR that corresponds to the hyperintense regions in the DWI, and hyper or isointense with respect to the normal white matter on FLAIR. At follow‐up, includes the hyperintense regions on FLAIR correspondent to the index stroke lesion identified at baseline. Regions of circular or ovoid shape, of 1‐2 mm diameter in size located inside these hyperintense regions, which are isointense with respect to the normal‐appearing white matter, were also included	*Properties*: Measured on the FA, MD and T1 maps after multiplying these parametric images by the correspondent binary masks *Volume*: Quantified from binary masks obtained semi ‐automatically on FLAIR by thresholding combined with a region‐growing algorithm and guided by the DWI image *Scores*: Coded following standard templates (Wardlaw and Sellar 1994; The IST‐3 Collaborative Group, 2015)
Old stroke lesions	Visible hyperintense regions on FLAIR and T2W MRI extending to the cortex, generally bordering a distinguishable region of low density with negative mass effect. In T1W they are seen as hypointense regions contiguous to the subarachnoid space or curvilinear hyperintensities signaling cortical laminar necrosis. Similar to the index stroke lesions at follow‐up, they included isointense regions (with respect to the white matter) of circular or ovoid shape, of 1–2 mm diameter in size located inside or bordering these FLAIR/T2W hyperintense regions	*Properties*: Measured on the FA, MD and T1 maps after multiplying these parametric images by the correspondent binary masks *Volume*: Quantified from binary masks obtained semiautomatically on FLAIR by thresholding combined with a region‐growing algorithm and guided by radiological knowledge *Scores*: Coded following standard templates(Wardlaw and Sellar 1994; The IST‐3 Collaborative Group, 2015)
New stroke lesion appearing during follow‐up	Ischemic: Hyperintense regions on the FLAIR images at follow‐up, outstanding for their size, location and shape after separately classifying the rest of the hyperintense regions. Could be proximal to a cavity or evident tissue loss. Hemorrhagic: Large T2*W hypointensity with irregular shape not present at baseline	*Properties*: Measured on the FA, MD and T1 maps after multiplying these parametric images by the correspondent binary masks *Volume*: Computed from binary masks semiautomatically obtained by thresholding and region‐growing on FLAIR (if ischemic) or T2*W (if hemorrhagic) *Scores*: Coded following standard templates (Wardlaw and Sellar 1994; The IST‐3 Collaborative Group, 2015)
Tissue loss due to stroke	CSF adjacent to the cortical stroke lesions (i.e., in old stroke lesions and index stroke lesions at follow‐up) when a visible tissue loss, compared with the contralateral hemisphere, was identified. This is labeled as “CSF due to old stroke” or “CSF due to index stroke at follow‐up”. In cases where visible ventricular or sulci enlargements made difficult to discern the extent of the tissue loss, this was annotated for further consideration in the analyses	*Volume*: computed from binary masks semiautomatically obtained by thresholding and region‐growing on FLAIR using Analyze^™^
Cavities (Lacunes)	Circular or ovoid hypointense regions on FLAIR, of more than 3 mm diameter, with intensities similar or equal to those of the CSF, located adjacent or surrounded by a hyperintensity identified as stroke lesion in the subcortical region, brain stem or cerebellar peduncles. These were assumed to be related to or in the stroke lesions, and were labeled “cavities in index stroke at follow‐up” or “cavities in old stroke”. When their size was equal to or less than 3‐mm diameter or their intensity was higher than that of the CSF, this was annotated for further consideration in the analyses. If the cavities of more than 3‐mm diameter were not related to a stroke lesion at baseline, they were disregarded. If they appeared at the follow‐up scan, they were separately assessed and labeled as “new cavity at follow‐up”	*Volume* and *count* obtained from binary masks generated semiautomatically from the Object Counter Tool in Analyze^™^, followed by a connected component analysis done in MATLAB R2014a *Scores*: Coded following standard templates (Wardlaw and Sellar 1994; The IST‐3 Collaborative Group, 2015)
Microbleeds	Small deposits of blood degradation products –mainly hemosiderin‐ contained within macrophages– are in close spatial relationship with structurally abnormal vessels (Martinez‐Ramirez et al. 2014). They appear as round hypointense dots on T2*W (Cordonnier et al. 2007)	*Count*: computed from shape (sphericity > 0.7), size and connected component analyses of regional iron deposits binary masks, done in MATLAB R2014a *Scores*: BOMBS (Cordonnier et al. 2009)
Iron Deposits	All hypointensity clusters on T2*W. Large and nonspherical clusters of T2*W hypointensities in the basal ganglia and substantia nigra, which correspond with isointense or hyperintense voxels on T1W, are separately noted. Possible cortical mineralization, which can be seen associated with old cortical strokes, is carefully excluded. T2*W hypointensities in the third ventricle and choroid plexus are also excluded under the assumption that these could be calcifications or a manifestation of a different pathology	Regional volume determined fully automatically from binary masks generated as per Glatz et al. 2015 (Glatz et al. 2015) in the deep gray matter structures and brainstem, and semiautomatically by thresholding (Valdes Hernandez et al. 2014) in the rest of the brain *Scores*: Mineral deposition load in basal ganglia rated from 0 (none) to much (4) (Penke et al. 2012)
Normal‐appearing white matter	Brain tissue that appears dark on T2W and FLAIR and with intensities from 50–75% of the maximum intensity value on T1W, coincident with the regions classed as white matter on the human brain atlas available at http://www.thehumanbrain.info/, after excluding stroke lesions and hyperintensities	*Properties*: Measured on the FA, MD and T1 maps after multiplying these parametric images by the correspondent binary masks *Volume*: Determined automatically by fusing the T1W and T2W sequences using MCMxxxVI followed by the removal of less‐intense WMH, and possible inclusion of some subcortical structures (see text)
Deep gray matter	Normal‐appearing caudate, putamen, globus pallidus, thalami and hippocampi	*Properties*: Measured on the FA, MD and T1 maps after multiplying these parametric images by the correspondent binary masks *Volume*: Obtained from binary masks determined automatically using FSL‐FIRST (Patenaude et al. 2011) followed by automatic subtraction of infarcts, lacunes and WMH, and manual editing when required
Hippocampi	Subcortical structures located bilaterally under the cerebral cortex in the medial temporal lobe, which are part of the limbic system	*Properties*: Measured on the FA, MD and T1 maps after multiplying these parametric images by the correspondent binary masks *Volume*: Determined automatically from binary masks obtained using FSL tools (SUSAN, FLIRT, FIRST) followed by manual editing when required *Shape*: 3D triangular mesh obtained automatically from the binary masks as per Kim et al. 2015 (Kim et al. 2015)
Contour rings in normal‐appearing WM	Concentric rings extended symmetrically from the WMH binary mask located in regions that intercept with the normal‐appearing white matter binary mask	Indexed map obtained by dilating the WMH mask at two consecutive distances of 2 mm each (Munoz Maniega et al. 2015)

**Figure 2:**
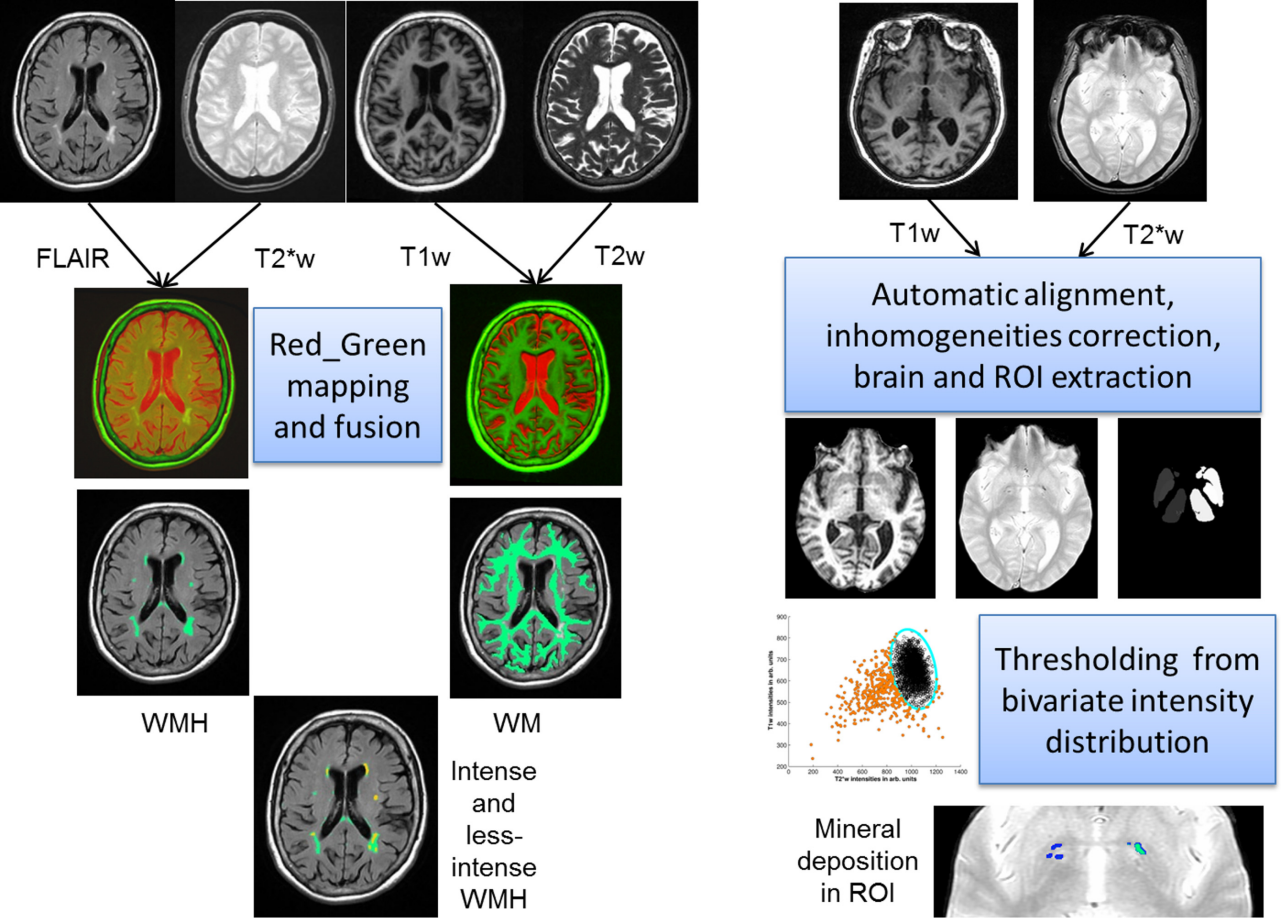
Diagram of the steps in the two structural automatic processing pipelines to assess normal tissues and WMH (left) using MCMxxxVI and regional mineral deposition (right). The top row shows the structural images that each pipeline uses. From top to bottom is a representation of each step's output. Relevant software are freely available (see text). WMH = white matter hyperintensities, WM = gross white matter directly obtained from the quantised color fusion of T2W and T1W, Intense and less‐intense WMH are obtained from voxel‐wise analysis of coincidence between WMH and WM binary masks, ROI = region of interest.

At baseline, the presence or not of the index stroke lesion is recorded. If the lesion is present, MRI modality in which it is visible, location, type, shape, maximum diameter (Del Bene et al. 2013), and whether it affects the cortex or not, according to standard templates (Wardlaw and Sellar 1994; The IST‐3 Collaborative Group, 2015) are also recorded. The presence, or not, of old infarcts and/or hemorrhages, their location and distribution (Wardlaw and Sellar 1994; The IST‐3 Collaborative Group, 2015) is also recorded. If the infarct is small subcortical, then the presence of cavitation (or not) is noted.

WMH are rated using Fazekas (Fazekas et al. 1988) and Wahlund (Wahlund et al. 2001) visual rating scores. For some analyses, a total Fazekas WMH score, ranging from 0 to 6, is obtained by summing the periventricular and deep white matter scores. We also visually assess WMH load by brain subregions: frontal, parieto‐occipital, temporal, infratentorial, and basal ganglia, according to Wahlund scores.

Perivascular spaces are rated using a scale developed in‐house (Potter et al. 2015), which can be accessed from: http://www.ed.ac.uk/polopoly_fs/1.163877!/fileManager/epvs-rating-scale-user-guide.pdf. This scale uses standard T2W structural brain MRI to assess the severity of the perivascular spaces located in three major anatomical regions: midbrain, basal ganglia, and centrum semiovale.

Microbleeds are rated using the Brain Observer MicroBleed Scale (BOMBS) (Cordonnier et al. 2009). The total number of definite microbleeds is determined using T2*W and susceptibility‐weighted images, and separately recorded for each brain hemisphere by brain regions: gray/white matter junction, deep white matter, basal ganglia, internal/external capsule, thalamus, and posterior fossa (Cordonnier et al. 2009). Basal ganglia iron deposition is rated from “none” (0) to “much” (4) (Penke et al. 2012). The presence of any arterial ectasia is recorded.

Atrophy (deep and superficial) is rated following a normative age template (Farrell et al. 2009), with superficial and deep coded separately ranging from none to severe in a scale from 1 to 6 according to the centiles into which the template is divided, being 1 (<25^th^), 2 (25–50^th^), 3 (50–75^th^), 4 (75–95^th^), 5 (>95^th^), and if ≫5, 6 is used. Alternatively, a “severe – moderate – mild – none” classification using standard examples can be used (The IST‐3 Collaborative Group, 2015).

From the follow‐up scans, in addition, we record atrophy progression, change in the number of microbleeds, the ratings of the iron deposition in the basal ganglia, the number of perivascular spaces, and WMH using the Prins visual ratings (Prins et al. 2004). We also record the status of the index stroke lesion: if cavitated, disappeared, increased, or decreased in size, and an estimate of its current maximal diameter. If there are new stroke lesions, we record the location, type, and whether they are already cavitated or not.

#### Computational assessment

All images are converted from DICOM to Analyze 7.5 format using software developed in‐house. All structural sequences obtained at baseline and follow‐up are rigidly and linearly registered to the T2W image of the baseline scan using FSL‐FLIRT (http://fsl.fmrib.ox.ac.uk/fsl/fslwiki/FLIRT) (Jenkinson et al. 2002). To guarantee the quality in the registration, given the high anisotropy of the voxels, it is done in two steps: (1) registration to the T2W image acquired at the same time point, (2) application of the matrix that defines the space transformation of the T2W image from follow‐up to baseline.

The measurements obtained computationally from the structural images are described in Table 2, performed separately per scanning session and blind to all other results; with the exception of the intracranial volume (ICV), which is performed only once at baseline and used for all the follow‐up analyses. The ICV is extracted semiautomatically using the T2*W GRE sequence, with the Object Extraction Tool in Analyze^™^ 11.0 followed by manual editing (Valdes Hernandez et al. 2012c). We then use MCMxxxVI (Valdes Hernandez et al. 2012c), a multispectral color fusion technique, to identify other intracranial tissues. This technique is implemented in www.sourceforge.net/projects/bric1936. It applies minimum variance quantization to a region (i.e., the ICV) of the color image obtained after fusing two or three coregistered MRI sequences, and mapping them in the red–green–blue color space (Hernandez et al. 2010). Cerebrospinal fluid, venous sinuses, choroid plexus, and meninges are considered together as “non‐brain” intracranial features, and extracted semiautomatically from the red/green color fusion of coregistered T2*W and FLAIR images using this technique. The resultant “non‐brain” binary masks are subtracted from the ICV to provide a measure of total brain tissue volume. The brain tissue binary masks are input to MCMxxxVI to define and extract all FLAIR hyperintensities on each dataset. These correspond to the regions in yellow color on the coregistered FLAIR and T2*W mapped in green and red, respectively. The software that implements this technique, generates binary masks of the FLAIR and T2W‐hyperintense regions, which are converted into object maps to be further separately classed as WMH, old stroke lesions, index stroke lesions, and new lesions appearing at follow‐up, using the Region of Interest tool in Analyze 11.0^™^ and definitions/criteria from Table 2. The tissue loss due to stroke and cavities is separately delineated on the FLAIR images by thresholding combined with a region‐growing algorithm, followed by manual editing. All assessments were checked by an independently trained image analyst and reassessed in case of errors. An experienced neuroradiologist also checked 20% of the computational measurements.

Normal‐appearing white matter is also extracted using MCMxxxVI (Valdes Hernandez et al. 2012a). However, to avoid including any subcortical gray matter structures as white matter, the following steps are sequentially implemented:

1. Noise reduction in the T1W image, achieved using the FSL tool SUSAN (http://fsl.fmrib.ox.ac.uk/fsl/fslwiki/SUSAN) that implements a nonlinear filtering combined with edge and corner detection for preserving the image leftacteristics. We use a Gaussian kernel of 3 × 3 × 3 mm operating in the 3D volume, a local median filter when single‐point noise is detected, and a brightness threshold equal to 10% of the robust range to preserve the edges (i.e., boundaries of each image feature).
2. Affine registration of the “clean” T1W image to an ageing brain template (already in standard space) using FSL‐FLIRT.
3. Model‐based subcortical segmentation of hippocampus, thalamus, putamen, globus pallidus, and caudate nucleus separately and in both cerebral hemispheres using FSL‐FIRST (http://fsl.fmrib.ox.ac.uk/fsl/fslwiki/FIRST) (Patenaude et al. 2011) optimized for the current imaging protocol and incorporating boundary correction in the hippocampus and in the caudate nucleus.
4. Erosion of the resultant probabilistic masks of the putamen, globus pallidus, and caudate nucleus, by a single voxel using a diamond‐shaped structuring element and binarization of the result.
5. Regeneration of a binary mask for the thalami using only the corresponding region with the highest probability within the probabilistic segmentation output obtained from FSL‐FIRST.
6. Generation of a binary mask with all subcortical regions combining the output of steps (4) and (5) (i.e., summing the corrected binary masks from all the structures).

The “corrected” white matter mask corresponds to the regions of the previously obtained white matter mask in which the output of step 6 was equal to zero. However, still in this white matter mask, pale and less‐intense FLAIR hyperintensities are included. They are separately classified as less‐intense WMH after examining the coincidence of these white matter masks and the WMH binary masks. The regions in which both masks coincide are labeled as less‐intense WMH: that is, regions hyperintense on FLAIR, but not hyperintense on T2W and not sufficiently hypointense on T1W. The regions in which the WMH mask does not coincide with the white matter mask are labeled as intense WMH: that is, regions hyperintense in both FLAIR and T2W and hypointense in T1W. The regions not included in the WMH mask, but included in the white matter mask are labeled as normal‐appearing white matter (NAWM). This “intense” and “less intense” subdivision is in agreement with findings from a postmortem study that showed a gradual difference in the severity of astrogliosis from well‐defined regions that corresponded to intense WMH → less‐intense WMH → NAWM on MRI (Gouw et al. 2008). More neuropathology studies are needed to determine the range of pathological correlates of WMH by degree of intensity on MRI.

Iron deposits are extracted using a fully automatic segmentation of the multifocal T2*W hypointensities in the subcortical structures and brain stem (Glatz et al. 2015) (https://github.com/aglatz/mineral-deposit-segmentation-pipeline/tree/master/libBRIC/mineral-deposit-segmentation), and a semiautomatic segmentation of those located elsewhere (Valdes Hernandez et al. 2011). Briefly, we perform a rigid body transformation to register the T1W volume to the T2*W using FSL‐FLIRT with the correlation ratio as optimization criterion. N4 (Tustison et al. 2010) is used to correct for nonanatomical intensity variations caused by multiple factors including inhomogeneous performance of the radiofrequency transmit and receive coils. Non‐brain structures visible on T1W volumes are removed by transforming the brain masks from T2*W to T1W space and applying them to the corresponding T1W volumes. Subcortical structures (including basal ganglia, brainstem, hippocampi, and thalami) and NAWM are segmented as explained above. The automatic delineation of T2*W hypointensities in the subcortical gray matter and brain stem is achieved with thresholds derived using an adaptive outlier detection method from the bivariate T2*W/T1W intensity distributions in each structure of the preprocessed T1W/T2*W images. Artifacts are reduced by filtering connected components in the binary masks of the T2*W hypointense clusters based on their standardized T2*W intensity variance and appearance on T1W MRI. The results of the automatic segmentation are individually checked by an experienced analyst blinded to any other imaging and patient information, and manually rectified if required. The semiautomatic segmentation of T2*W hypointensities in other parts of the brain is performed using the “Object Counter” module in Analyze^™^ 11.0 following the process described in (Valdes Hernandez et al. 2014). A slice is selected where the T2*W hypointensities appear, ideally with a variety of shapes and intensities to adjust the intensity threshold, starting from zero to less than half of the median intensity value of the NAWM. An estimated maximum and minimum size of the hypointense “objects” is then adjusted interactively. Binary masks of regional iron deposits are obtained from the intersection between the binary masks obtained from this semiautomatic process and the regional regions‐of‐interest and tissue masks.

Quantitative T1 maps are obtained by coregistering the 2° and 12° FSPGR sequences using FSL‐FLIRT, with T1 being determined from:

$$\frac{1}{T1}\;=\;\frac{1}{TR}\;\ln\;\left[\frac{S_{R}\sin\;\alpha_{2}\;\cos\;\alpha_{1}\;-\;\sin\;\alpha_{1}\;\cos\;\alpha_{2}}{S_{R}\sin\;\alpha_{2}\;\sin\;\alpha_{1}}\;\right]$$

where *TR* is the repetition time (= 8.2 ms), *α*_1_ = 2°, *α*_2_ = 12°, *S*_*R*_ = *S*_*1*_/*S*_*2*_, and *S*_*1*_ and *S*_*2*_ the signal intensity values in each voxel for the 2° and 12° FSPGR images, respectively (Wardlaw et al. 2011).

#### Evaluation of measurements’ accuracy and interanalyst agreements

All quantitative methods have been previously evaluated (Hernandez et al. 2010; Valdes Hernandez et al. 2011, 2012a,b,c). On a sample of 150 individuals aged 71–72 years, the mean difference between a reference standard manually outlined by an experienced analyst and the measurements of ICV performed with the method described in this protocol was 2.7% (95% CI ±7%)(Valdes Hernandez et al. 2012c). In test–retest analyses for accuracy of measuring WMH volume on 14 datasets from volunteers aged 65–70 years and patients with mild nondisabling stroke aged 50–90 years, MCMxxxVI had a coefficient of variation for repeated measurements of 0.21 (Hernandez et al. 2010). On a subsequent test on a sample of 20 septuagenarian individuals, selected to represent the whole range of WMH load, the spatial agreement (i.e., average Jaccard Index) with manual reference segmentations was 0.98 (i.e., almost perfect) (95% CI = ±0.03) for white matter, 0.46 (i.e., substantial) (95% CI = ±0.12) for cerebrospinal fluid, and 0.61 (i.e., substantial to almost perfect) (95% CI = ±0.37) for WMH (Valdes Hernandez et al. 2012a).

**Figure 3:**
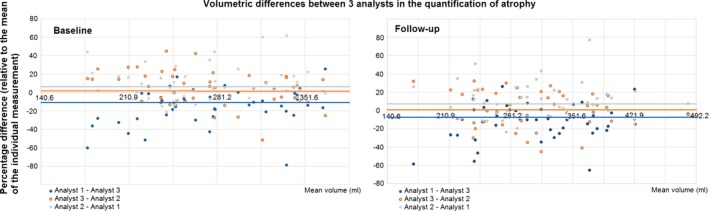
Bland–Altman plots showing the interobserver reliability in the semiautomatic segmentation of non‐brain tissue volume, obtained from measurements done by three analysts using MRI scans from 45 stroke patients. Visual analysis of the results showed that partial volume effects were the main cause of discrepancy between analysts. Horizontal lines indicate mean interanalysts differences.

Given: (1) the slice thickness of this protocol's sequences and its adverse influence on general atrophy measurements (i.e., due to partial volume effects), (2) the fact that the multispectral threshold for separating brain tissue from non‐brain is interactively decided by the image analyst (i.e., analyst dependent), and (3) the relatively lower precision (compared to the ones obtained for segmenting ICV, normal white matter, and WMH) that our multispectral method had on segmenting cerebrospinal fluid; we evaluated the interanalyst agreement in quantifying the general atrophy (i.e., non‐brain tissue within the ICV) on a subsample of 45 stroke patients. We analyzed segmentation results generated by three analysts done blind to each other. The interanalyst agreement was excellent (mean dissimilarity index: 0.3 (baseline) and 0.28 (follow‐up) with slopes of −3.5 × 10^−6^ and −7.03 × 10−^6^ mL, respectively) (Appendix S1). The interanalyst differences in the volumetric measurements were also excellent with levels of 4.4% (SD 18.7%) of the volume measured (mean 252.6 mL, SD 59.7 mL) at baseline and 1.4% (SD 19.7%) of the volume measured (mean 285.7 mL, SD 70.3 mL) at follow‐up between analysts with similar experience. As expected, these were slightly higher when analysts had different levels of experience (Fig. 3). The influence that these interobserver differences had on the longitudinal measurements were: mean interanalyst difference, 17.8% (SD 145.2%) of the volume measured (mean 33.8 mL, SD 23.3 mL).

### Selection and extraction of Regions of Interest

**Figure 4:**
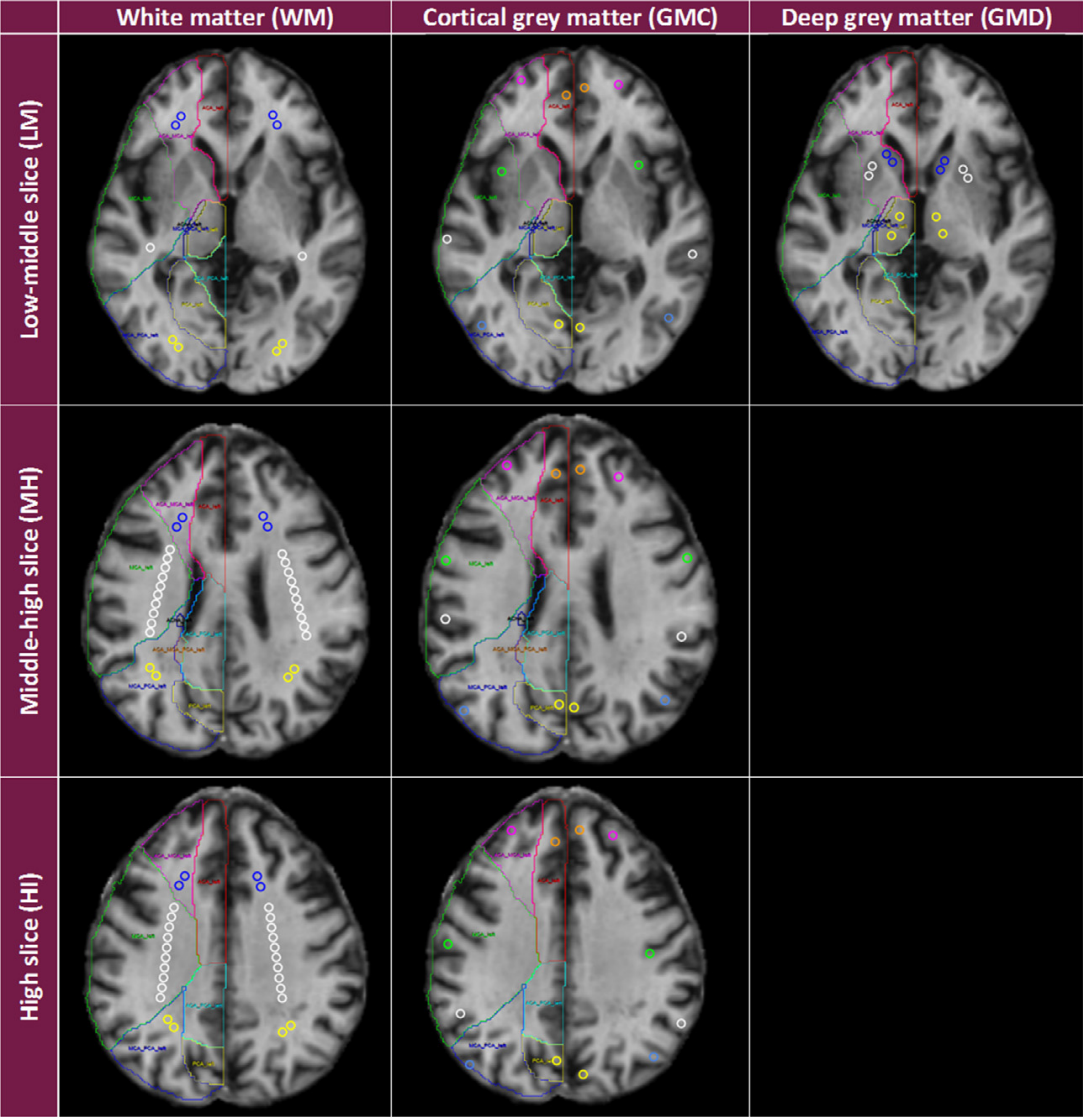
Regions of Interest (ROI) template, adapted from Wardlaw et al., Stroke 2008. Axial fast spoiled gradient echo slices acquired with flip angle = 12°, showing the ROI object maps corresponding to normal‐appearing cortical and subcortical gray and white matter, and the corresponding arterial territories, on the base‐to‐middle, middle‐to‐top, and top slices. ACA = anterior cerebral artery arterial territory, ACA_MCA = border‐zone (watershed) area between anterior and middle cerebral arteries, GM = gray matter, GMC = cortical gray matter, GMD = deep gray matter, HI = high slice, LM = low‐middle slice, MH = middle‐high slice, PCA = posterior cerebral artery, MCA_PCA = border‐zone (watershed) area between middle and posterior cerebral arteries, ROI = regions of interest, WM = white matter. WM ROIs (anterior to posterior) (1) blue ROIs are in the anterior part of the frontal lobe in the ACA_MCA between the ACA and the MCA; (2) white ROIs are in the MCA arterial territory (in the corona radiata in MH and Hi slices); (3) yellow ROIs are in the MCA_PCA between the MCA and the PCA. GMC ROIs: (1) orange ROIs are in the medial frontal gyrus along the longitudinal (i.e., mid‐central) cerebral fissure in the ACA arterial territory; (2) pink ROIs are near the superior frontal ACA_MCA; (3) green ROIs are in or near the precentral gyrus near the precentral sulcus (or near the short gyri of insula near the central sulcus of insula in the LM slice) in the MCA arterial territory; (4) white ROIs are in or near the postcentral gyrus near the postcentral and lateral sulci in the MCA arterial territory; (5) blue ROIs are in or near the angular gyrus near the intraparietal sulcus (or the inferior temporal gyrus in the LM slice) in the MCA_PCA; (6) yellow ROIs are near the cuneus along the longitudinal cerebral fissure in the PCA arterial territory. GMD ROIs: (1) blue ROIs are in the caudate heads; (2) white ROIs are in the putamen (lentiform nuclei); (3) yellow ROIs are in the thalami. Source: created by Miss. Linda Viksne (see Acknowledgments), using vascular maps from Hoban et al., HBM 2014.

Regions of interests (ROIs) from “pure” tissue types and cerebrospinal fluid are obtained from a modification to an existent template (Wardlaw et al. 2008) to measure quantitative parameters (i.e., FA, MD, and T1) on specific brain regions. The ROI placements complement the tissue segmentation as the presence of partial volume effects (particularly in the slice direction) could affect quantitative measurements using the tissue masks obtained from the pipelines described above. This new template also guarantees consistency in the placement of ROIs across subjects and across studies so as to reduce confounds that variations on their location could pose to the analyses. It includes circular ROIs of 15 mm^2^ (approximately) placed on representative axial slices at the base, base‐to‐middle, middle‐to‐top, and top of the brain (Viksne et al. 2015). Each ROI, with a volume of 45–50 mm^3^, is enumerated so as to provide information on specific locations (Fig. 4). Typical brain arterial territories (Hoban et al. 2014) also appear on the template.

In addition, to assess how the proximity of the WMH affects the NAWM integrity, we automatically generated ROIs at a range of distances from the WMH by dilating the WMH masks in increments of two voxels (about 1.88‐mm wide) up to a distance of about 10 mm, followed by subtracting, from each dilated ROI, the previous one, that is, the WMH mask was subtracted from the “2‐mm” ROI, the 2‐mm ROI subtracted from the 4‐mm ROI and so on, so only the surrounding contours remained (the distances quoted are approximate as they are limited by finite voxel size). To avoid running into tissues other than NAWM, we keep only the ROI contour voxels that intersect with the NAWM mask. For each patient, we calculate the quantitative parameters (i.e., FA, MD, T1) within each ROI, as well as within the remaining NAWM and analyze these data (adjusted by age, gender, and other factors as appropriate) for each distance to assess the spatial relationship in relation to “pure” WMH (Munoz Maniega et al. 2015).

### Diffusion‐weighted imaging analysis

From the diffusion‐weighted images, parametric maps of fractional anisotropy (FA) and mean diffusivity (MD) maps are generated. For each dataset, nonlinear registration (Modat et al. 2010) is used to align the tissue masks at baseline in the structural space (T2W) with the parametric maps in the diffusion space. We apply NiftyReg from http://sourceforge.net/projects/niftyreg/ using TractoR (http://www.tractor-mri.org.uk/diffusion-processing) to obtain the transformation between the structural T2W brain image and the averaged diffusion volume with b = 0 s/mm^2^. To avoid partial volume averaging with CSF due to registration inaccuracies, the CSF mask is dilated by one voxel in each direction and then subtracted from the NAWM, gray matter, and WMH masks in the diffusion space. After registration, the binary intense and less‐intense WMH, deep gray matter and NAWM binary masks (definitions in Table 2) are used to obtain averaged FA and MD values for these tissue types in each subject. Averaged FA and MD values are also obtained from the ROIs placed following the template referred above as appropriate.

### Spatial distributional analyses

To generate spatial distribution probability maps of each imaging SVD marker, (e.g., WMH, lacunes, stroke lesions, etc.) for different groups of patients, we first map all sequences to the T1W sequence and to age relevant templates (Farrell et al. 2009) in standard space, using affine transformation in FSL‐FLIRT. These templates are publically available in the BRAin Images of Normal Subjects (BRAINS) image bank (http://www.sinapse.ac.uk/research-resources/brains-project). Then, we use the transformation matrices obtained, to map the lesions/tissue masks to standard space using the same tool. Subsequently, groups of binary masks in standard space are averaged to generate the spatial distribution probability maps of each SVD imaging marker.

It is important to highlight that affine and nonlinear registration distort the actual volume of the lesions, and therefore these probability distribution maps are only for studying the relative spatial distribution of the SVD markers between and within patient groups, not for measuring lesion size. The mathematical demonstration of how and why this distortion occurs appears in the Appendix S2. To illustrate with an example, we delineated the index stroke lesion as per this protocol (Table 2) on brain MRI datasets from 187 patients who presented with acute lacunar stroke symptoms and had a recent small subcortical infarct confirmed on MR diffusion imaging (Valdes Hernandez et al. 2015). Then, we mapped the delineated lesions on a common “standard” space, and measured the lesion volume before and after the space transformation. Lesion volumes after the space transformation were 2.5 times bigger (IQR 0.67) than when measured in their native space, thus representing a median increase in 1.36 times (IQR 0.12) per dimension (see comparative graph in Appendix S2).

### Correspondence between computational volumetric measurements and visual rating scales

**Figure 5:**
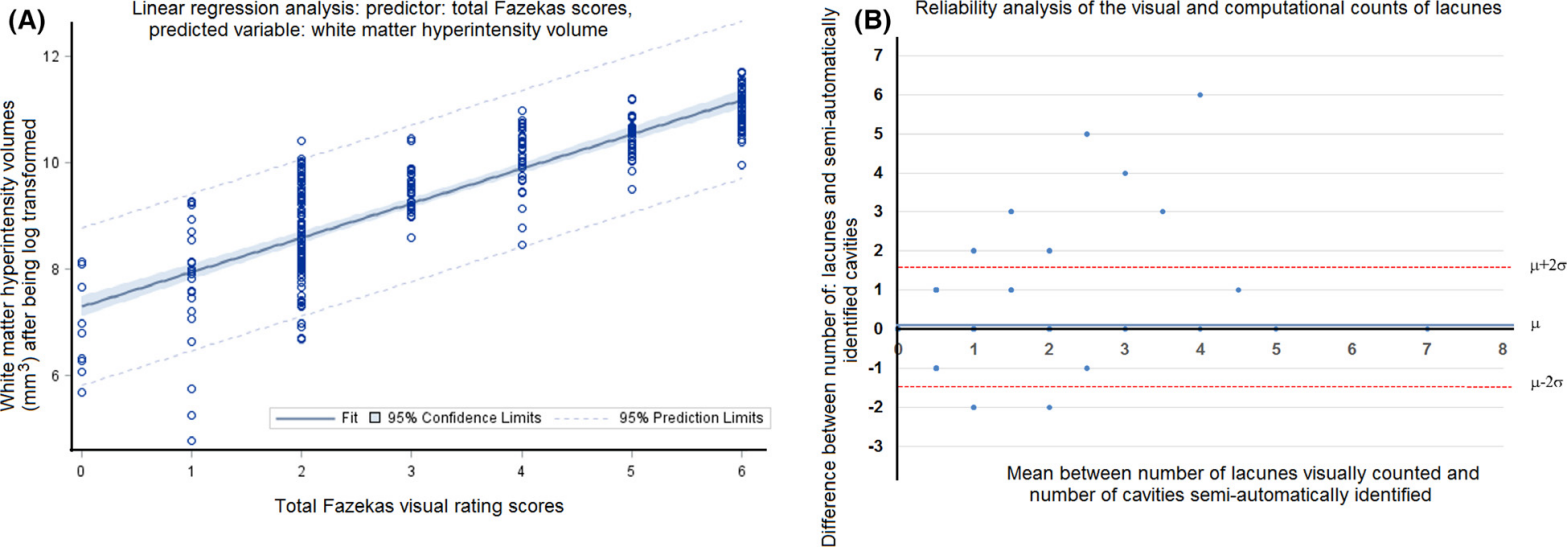
Computational versus visual rating assessments obtained from a sample of 206 mild stroke patients used to develop the present protocol. (A) Univariate linear regression results between the log transformed values of the WMH volumes and total Fazekas scores: an increase of 1 in the total Fazekas score represented an increase of 7.3 mL (95% CI [7.1 7.5], *P* < 0.001), and (B) Bland–Altman analysis evaluating agreement between computational cavity count and radiological identification of lacunes: mean difference between assessments = 0.19 lacunes ± 0.81. The initial correspondence between Fazekas scores and WMH volume was nonlinear throughout the score, but with the log transformed values this correspondence was linear. This was checked by standard statistical fit diagnostics.

The correlation between the WMH volumes resulting from the MCMxxxVI method and Fazekas scores, determined on a sample of 662 older community‐dwelling individuals of mean age 72.8 years, was high (Spearman's *ρ *= 0.78, *P* < 0.001) (Valdes Hernandez et al. 2012b). Discrepancies were caused by: 1) subtle WMH identified visually but omitted from the volume, 2) prominent ventricular caps but thin body lining giving a periventricular score of 1 or 2 but large WMH volume, and 3) small deep focal lesions that increase the score disproportionately when beginning to coalesce with little change in WMH volume (Valdes Hernandez et al. 2012b). We did similar analysis using a sample of 206 mild stroke patients. Periventricular and deep scores were summed to a total score of 0–6 and showed a highly significant correlation with WMH volume (Spearman's *ρ *= 0.88, *P* < 0.001). In addition, we used linear regression to estimate the strength of this association: an increase of 1 in Fazekas score represented an increase of 7.3 mL (95% CI [7.1 7.5], *P* < 0.001) (Fig. 5A) in WMH volume. We also evaluated the agreement between the count of round or ellipsoidal “cavities” of 3–7 mm maximum diameter detected semiautomatically (considered to correspond to lacunes) and the number of lacunes reported from the visual assessment, using Bland–Altman analysis (Fig. 5B). The mean difference between the assessments was 0.19 (SD ± 0.81).

## Discussion

We present a comprehensive approach to leftacterize cross‐sectional and longitudinal neuroimaging biomarkers of SVD, which aims to operationalize the STRIVE recommendations. The proposed workflow is adequate to higher resolution images, including those acquired at 3T, despite being applied to and evaluated on scans acquired at 1.5T. It is also suitable for studies of SVD (not necessarily stroke) and stroke in general as results from previous evaluations (cited) show. Recommendations in the sequences’ acquisition at higher field strengths are given. Additional methods may be needed for specific purposes (e.g., if dynamic contrast enhanced or dynamic susceptibility contrast sequences to study blood–brain barrier permeability or perfusion are added).

Stroke images are complex. The presence of multiple features with huge variation in distribution, size, and morphology pose a major challenge for computational analyses. The hybrid approach presented here optimizes accuracy of tissue segmentation and feature leftacterization using conventional MR images commonly acquired on clinical studies. Much of the image data used are routine clinical data. The differential leftacterization of each SVD feature, addressed by this protocol, is important. In the population, this protocol is designed to study WMH of uncertain origin coexist and sometimes coalesce with FLAIR and T2W hyperintense regions caused by stroke, and which are carefully separated by our computational segmentation protocol. If the latter are not distinguished from the total WMH burden, the WMH volume could increase by 20% or more, and brain volume loss could change by approximately 22 mL (i.e., reported as 1.65% of the average brain volume) (Wang 2013). Errors of these magnitudes in randomized drug trials could cause potentially effective treatments to be missed and ineffective treatments appear beneficial. In observational epidemiology, failure to differentiate cortical infarcts from WMH could make it look like WMH had increased or decreased more than they actually had and inflate apparent associations with thromboembolic stroke risk factors.

Computational methods for measuring brain structure and disease state offer quantitative measurements useful for several analyses, for example, atrophy and lesion burden. On the other hand, visual rating scales, despite their known inter‐ and intraobserver differences, ceiling, and floor effects (Cordonnier et al. 2009; Valdes Hernandez et al. 2012b; Potter et al. 2015), are sensitive to qualitative disease indicators that are difficult to assess computationally if present, for example, microbleeds, perivascular spaces. They could be the choice by excellence for larger multicenter studies in the absence of the availability of computational power. Our protocol combines widely used visual rating procedures with carefully tested computational methods to overcome the difficulties inherent in both types of techniques: computational and visual.

It has been widely acknowledged that existing fully automatic methods, developed using isotropic high‐resolution MR images of individuals with minimal or absent pathology, are not easily translated to routine MRI of individuals with a medium to large burden of mixed pathology, especially stroke patients (Wardlaw et al. 2015). We evaluated different fully automatic state‐of‐art frameworks that use machine learning techniques (Kloppel et al. 2011; Ithapu et al. 2014; Leite et al. 2015) or supervised classifiers (Anbeek et al. 2004) seeking to minimize observer input in the assessment of T2W/FLAIR hyperintensities. These frameworks, with variations, have been proposed to be suitable for studies of stroke (Mitra et al. 2014; Maier et al. 2015). However, the results from these methods are dependent on the training set. Given the variability in the pathology represented on the sample used to develop this protocol and its relatively small size (i.e., 250 patients), we cannot recommend them as alternative methods to substitute the validated ones presented in this protocol.

Reported mean intra‐ and interscanner coefficients of variation in automatic volumetric measurements of brain structures range from 0.87% to 15.1% (median 4.80%) (Huppertz et al. 2010). Aware of the influence that inter‐/intraobserver differences can have on volumetric measurements, our protocol uses fully automated pipelines where possible to maximize the use of state‐of‐the‐art automatic tools, but also carefully manual editing following validated procedures. This final step aims not only to remove artifacts that mimic true abnormalities (Wang 2013), but also to differentiate confounding features, for example: small punctate WMH in the deep white matter from perivascular spaces and these from lacunes (Valdes Hernandez et al. 2013). To raise awareness of the time and effort required to manually edit each structure/tissue/lesion, we present this information based on our experience, of applying this protocol, or parts of it, to images from more than 1200 individuals with SVD (Table 3). We anticipate that while improvements to speed up the processing are ongoing by many groups, this current information could contribute to future planning and execution of similar projects, and be of use to funding bodies and project managers.

**Table 3:** Estimated time per brain to perform the semiautomatic assessments, with individual's level of experience

Biomarker to segment	Estimated time per brain (mins)	Rater's experience (no. of datasets)	Biomarker to segment	Estimated time per brain (mins)	Rater's experience (no. of datasets)	Biomarker to segment	Estimated time per brain (mins)	Rater's experience (no. of datasets)
Hippocampi	20	>100	Ventricles	20	>100	ICV	20	>100
	15–20	>100		10–15	>100		15	>100
	15–20	<50		30	50–99		20	>100
				30	<50		20	>100
							20	>100
							15	<50
WMH/WM	30	>100	CSF	10	>100	Preprocessing	15	>100
	15–60	>100		15	>100		10	>100
	30	>100		5	>100			
	20–45	>100						
	15–30	>100						
	60	<10						

This information was collected from nine analysts with experience on the image analysis tasks performed on the datasets. Each analyst reported the estimated time that takes for him/her to manually rectify the boundaries of the “biomarker to segment”, and the (also estimated) number of datasets on which he/she did the referred assessment in the way this protocol describes.

While we use state‐of‐the‐art nonlinear registration techniques to map the native space into the diffusion space to measure diffusion parameters in the tissues and features, we use linear registration to distributional lesion maps. This is because it is not known whether the morphological deformations that stroke and SVD cause to the brain parenchyma reflect true displacements of the white matter fibers or whether these should be considered tissue loss that destroys neural tissue or forces neural tracts to be rerouted, redefined, or a mixture of all. Each scenario imposes different constraints on the warping algorithm, manipulated by the nonlinear registration technique, and influences the way structural images are deformed. This is the subject of ongoing work. Giving the complexity of the image contents, perhaps this is an area where machine learning approaches, spatial 3D modeling and more detailed serial imaging would be better at than the more traditional approaches to date.

## Conflict of Interest

None declared.

## Supporting information


**Appendix S1**. Evaluation of accuracy on brain tissue atrophy measurements.

**Appendix S2**. Analysis of the differences between volumetric measurements in native and standard spaces.
