# Supplementary material for: Rationale, design and methodology of the image analysis protocol for studies of patients with cerebral small vessel disease and mild stroke
Source: Brain Behav. 2015 Nov 26;5(12):e00415. doi: 10.1002/brb3.415 (PMC4714639; doi:10.1002/brb3.415)
Supplement: Supplementary file 1 — Appendix S1. Evaluation of accuracy on brain tissue atrophy measurements. [file BRB3-5-e00415-s001.docx]

**Rationale, design and methodology of the image analysis protocol for studies of patients with cerebral small vessel disease and mild stroke**

**Supplementary Material 1: Evaluation of accuracy on brain tissue atrophy measurements**

**1.1. Materials and methods**

Using MCMxxxVI as shown in Figure 2 of the main text, three analysts segmented the “non-brain” tissue (i.e. voxels on tones of red on the fused T2*-weighted/FLAIR after being respectively mapped in the red/green colour space) on 45 image datasets. Two analysts had similar levels of experience.

The following parameters were calculated at baseline and follow-up:

1. Dissimilarity index (DI): Determined using the following expression:

$$DI= \left\{ \begin{matrix} 1-\frac{RS\cap M}{RS\cup M} & \forall RS,M\in\boldsymbol{R}^{3} & RS>0 ;M>0 \\ 0 & \forall RS,M\in\boldsymbol{R}^{3} & RS=M=0 \\ 1 & \forall RS,M\in\boldsymbol{R}^{3} & RS=0 ; M>0 or RS>0 ;M=0 \end{matrix} \right. (S1\_1)$$

where RS is the binary mask that produced the measurement considered reference standard (generated from Analyst 2) and M is the binary mask that is evaluated (generated by Analyst 1 and Analyst 3). Values near 0 indicate few to none dissimilarities between RS and M, in other words: excellent agreement. Values of DI approach 1, when dissimilarities between both (i.e. RS and M) are considerable.

1. Dice coefficient or similarity index: Commonly used to evaluate image segmentation methods. It is a special case of Kappa coefficient and follows the same criteria to evaluate the strength of the agreements: 0 to 0.2 poor, 0.21 to 0.4 fair, 0.41 to 0.6 moderate, 0.61 to 0.8 substantial and 0.81 to 1.0 almost perfect. Following the same notation as Equation (S1_1), we express it mathematically as:

$$Dc= \frac{RS\cap M}{\frac{1}{2}\left( RS+M \right)} (S1\_2)$$

A value of 0 indicates no overlap between RS and M while a value of 1 indicates perfect agreement. In other words: higher numbers that are in the vicinities of 1 indicate that M matches the reference standard segmentation.

1. True positive fraction: Expresses the proportion of the positives that was correctly identified:

$$TPF= \frac{RS\cap M}{RS} (S1\_3)$$

2. True negative fraction: Expresses the proportion of correctly identified negatives.

$$TNF= \frac{\sim\left( RS\cup M \right)}{\sim RS} (S1\_4)$$

From the parameters determined in equations S1_3 and S1_4, the error rates can be calculated. The false positive rate equals 1-TPF and the false negative rate equals 1-TNF.

**1.2. Results**

Table S1_1 shows the values of the similarity measures that reflect the accuracy of the atrophy measurements.

Table S1_1. Accuracy of the atrophy measurements provided by Analyst 1 and Analyst 3 with respect to Analyst 2. Magnitudes are given in: average (standard deviation).

|  | Atrophy measurements from Analyst 1 | | Atrophy measurements from Analyst 3 | |
| --- | --- | --- | --- | --- |
|  | Baseline | Follow-up | Baseline | Follow-up |
| Volume (ml) | 255.67 (60.01) | 287.73 (70.56) | 291.42 (50.86) | 312.26 (65.74) |
| Dissimilarity index | 0.26 (0.09) | 0.26 (0.09) | 0.27 (0.08) | 0.27 (0.08) |
| Dice coefficient | 0.85 (0.07) | 0.85 (0.06) | 0.84 (0.06) | 0.84 (0.06) |
| True positive fraction | 0.82 (0.11) | 0.82 (0.11) | 0.87 (0.09) | 0.85 (0.11) |
| True negative fraction | 0.99 (0.002) | 0.99 (0.003) | 0.99 (0.005) | 0.99 (0.004) |

As can be appreciated from Table S1_1, similarity measurements at baseline and follow-up were almost equal. The misclassified voxels were minimal (e.g. true negative fraction nearly 1). The main source of disagreement were due to partial volume effects, manifested when deciding whether to include certain group of voxels or not. This is also reflected in the difference between the volumetric measurements between analysts 1 and 3.

Figure S1_1 shows the dissimilarity index obtained from baseline and follow-up measurements plotted vs. the average value between the reference measurement provided by a trained analyst and the measurement obtained from two other analysts. Although, as expected, slightly better agreement was obtained for higher volumes, the slope that reflects this tendency (represented in dotted lines in the graphs) was in the order of 10^-6^ volumetric units. (Figure S1_1).


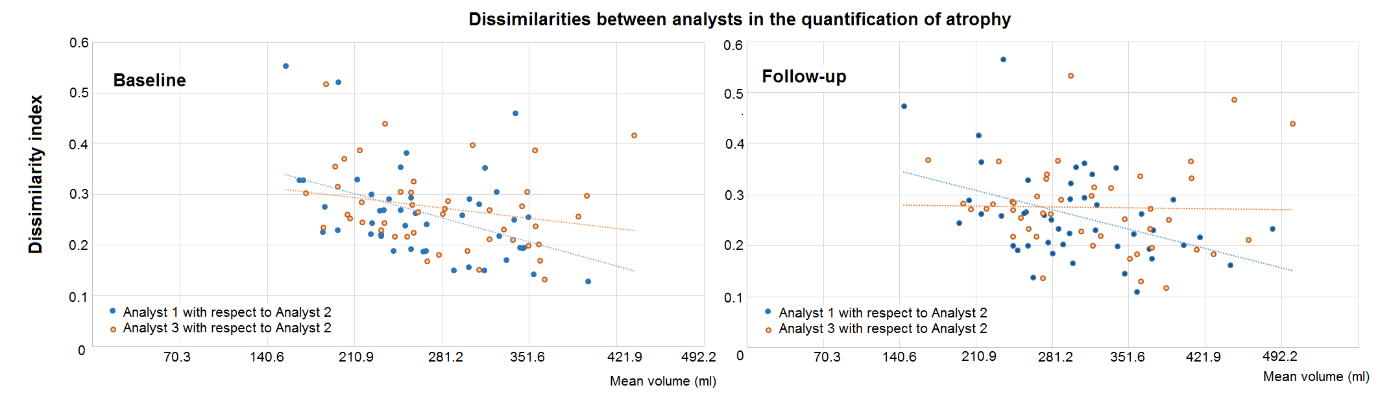


Figure S1_1. Plot of the dissimilarity index between atrophy measurements provided by two analysts (i.e. Analysts 1 and 3) and reference measurements (provided by Analyst 2) on 45 stroke image datasets.

**Reference**

Valdés Hernández MC, Gallacher PJ, Bastin ME, Royle NA, Muñoz Maniega S, Deary IJ, Wardlaw JM. Automatic segmentation of brain white matter and white matter lesions in normal aging: comparison of five multispectral techniques, Mag. Res. Imaging, 2012; 30(2):222-9 <http://www.sciencedirect.com/science/article/pii/S0730725X11003481>.
