# Supplementary material for: Rationale, design and methodology of the image analysis protocol for studies of patients with cerebral small vessel disease and mild stroke
Source: Brain Behav. 2015 Nov 26;5(12):e00415. doi: 10.1002/brb3.415 (PMC4714639; doi:10.1002/brb3.415)
Supplement: Supplementary file 2 — Appendix S2. Analysis of the differences between volumetric measurements in native and standard spaces. [file BRB3-5-e00415-s002.docx]

**Rationale, design and methodology of the image analysis protocol for studies of patients with cerebral small vessel disease and mild stroke**

**Supplementary Material 2: Analysis of the differences between volumetric measurements in native and standard spaces**

**2.1. The space transformation**

Aligning and rescaling individuals’ images to a standard size and orientation is a necessity for studying the progression and distribution of features – pathological and non-pathological- (e.g. white matter hyperintensities, microbleeds, stroke lesions) across a population. For this purpose, among the several types of existing space (i.e. coordinate) transformations (Maintz and Viergever, 1998), the rigid 3D affine is preferred. When comparative morphometry and group analysis of functional and physiological data require co-registration to establish correspondences across the anatomical structures, non-rigid transformations are preferred (Klein et al., 2009). But even such techniques require, prior to the warping, an affine transformation that rigidly aligns and rescales the patients’ image data. The affine space transformation preserves points, straight lines and planes, and can include translation, scaling, homothety, similarity transformation, reflection, rotation, shearing and combinations of them in any sequence.

**2.2. Mathematical analysis using basic algebra and geometry**

Mathematically, an affine transformation can be represented as the composition of two functions: a translation and a linear map. Thus, if the linear map that defines this transformation is represented as a multiplication by a matrix *A* and the translation as the addition of a vector
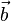
, an affine map
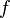
acting on a vector
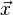
can be represented as:


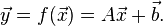
 (S2_1)

Equation (S2_1) is explained in details in <https://en.wikipedia.org/wiki/Affine_transformation>. Figure S2_1 illustrates the affine mapping of an image (represented by a blue square) initially on the coordinate space X, to the coordinate space Y. The two orange points on the vertices of each square illustrate the points *x_1_* and *x_2_* of the vector
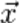
 if in the native (i.e. original) coordinate space, as they are transformed to
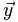
. The matrix A representing the space transformation is obtained, in the example, by sequentially multiplying the rotation, scaling and translation matrices defined as per above in the same figure.

**
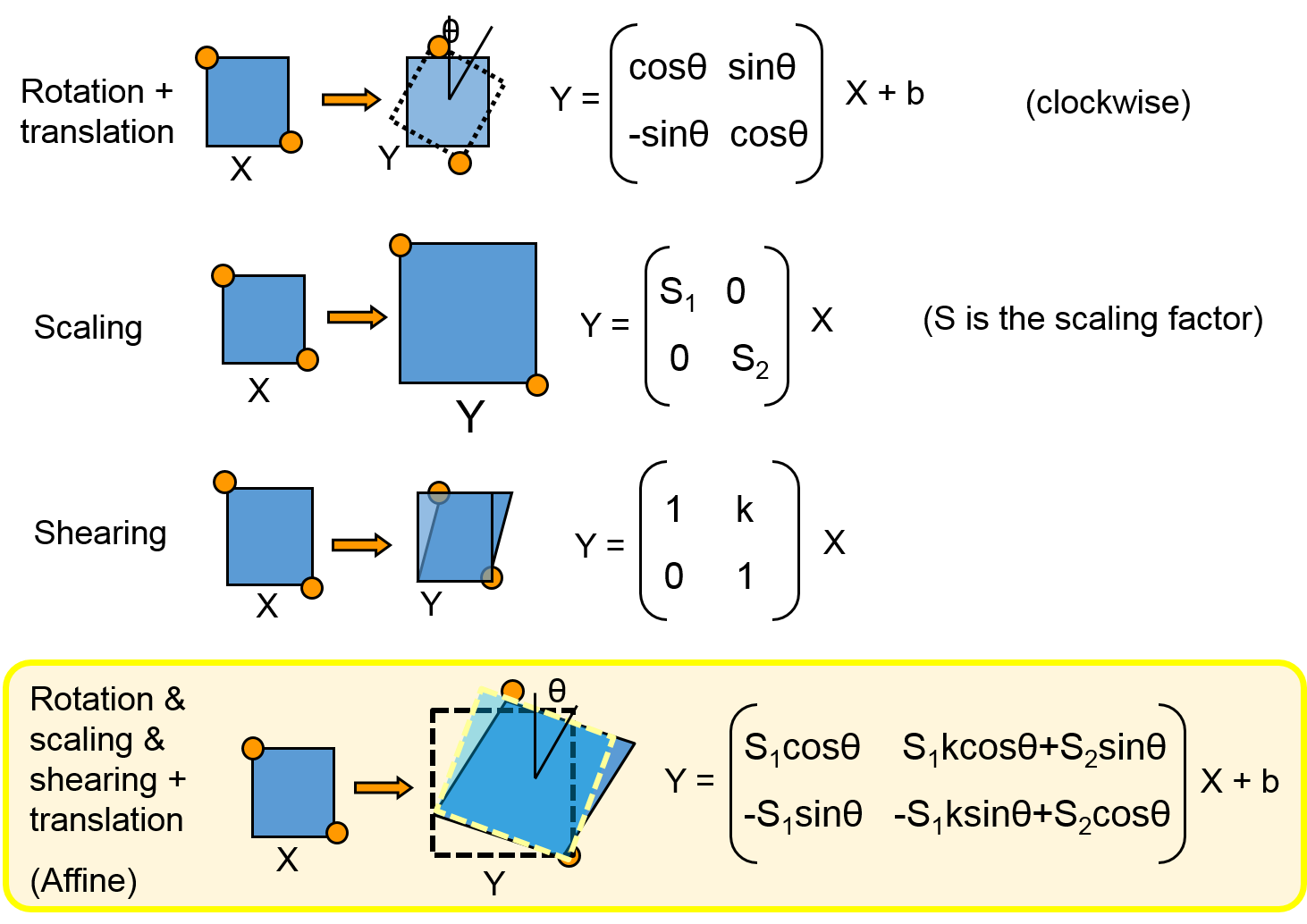
**

Figure S2_1. Illustration of the three main operations involved in an affine transformation. The combination of the space transformations represented in the three first rows (from top to bottom) results in an affine transformation as presented in the box highlighted in yellow. The resultant matrix can be corroborated by multiplying the rotation, scaling and shearing matrices shown above in this order. Notice that, as the matrix multiplication is non-commutative, a different transformation matrix will be obtained if the order in which the three matrices that intervene in the final result is altered.

As Figure S2_1 illustrates, rotations and translations do not alter the relative positions of the two orange points in X when they are transformed to Y. However, this is not the case when scaling and shearing are part of the space transformation. Figure S2_2 illustrates a practical example of MR image alignment where an individual’s brain needs to be enlarged to match the size of the brain used as reference.


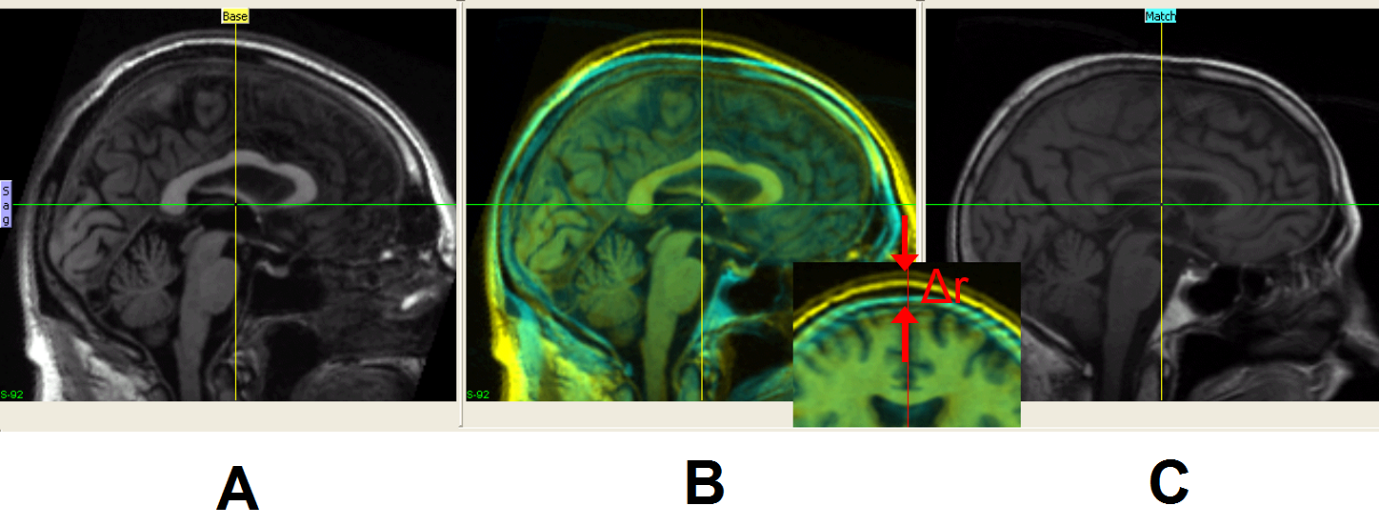


Figure S2_2. Example showing the midsagittal representation of a brain (C) when is rigidly aligned to a reference brain (A). Panel B shows the superposition of the two brains. In addition to differences in shape, there is a difference in size between both brains, clearly shown in the coronal view (inset).

As Figure S2_2 shows, for the individual’s brain (C) to match the size of the reference brain (A), the former needs to enlarge Δr units. To analyse the effect of this enlargement on specific brain features, let’s model the brain as a sphere and then pass a plane through the sphere as Figure S2_3 shows.


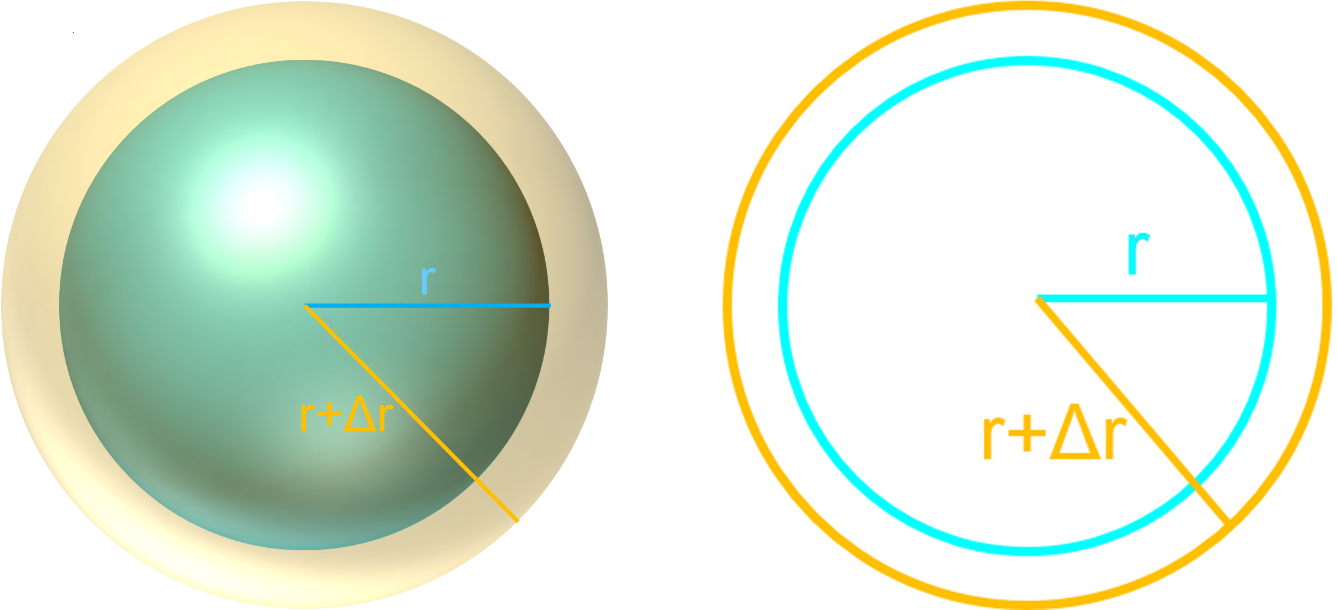


Figure S2_3. Diagram representing two concentric spheres with different radiuses, and the plane dividing each sphere in two identical halves. The reference radius corresponds to the blue (inner) sphere.

In Figure S2_3, we have the blue circumference representing the border of the individual’s brain and the yellow circumference representing the border of the reference’s brain. The perimeter of the blue circumference is equal to 2πr and that of the yellow is 2π(r+Δr). Equally, the area of the blue circumference is πr^2^ and that of the yellow is π(r+Δr)^2^. Removing the parentheses, we have:

$reference^{'}s perimeter 2\pi\left( r+\Delta r \right)=2\pi r+2\pi\Delta r$ (S2_2)

$reference^{'}s area \pi\left( r+\Delta r \right)^{2}= \pi r^{2}+2\pi r\Delta r+\pi\Delta r^{2}$ (S2_3)

The first term of equation (S2_2) is equal to the perimeter of the blue circumference (i.e. individual’s perimeter) and the first term of equation (S2_3) is equal to the area of the blue circumference (i.e. individual’s area). Therefore, the rest of the terms in equations (S2_2) and (S2_3) are the increments in perimeter (i.e. 2πΔr) and area (i.e. 2πrΔr+πΔr^2^) of the reference (i.e. yellow circumference) with respect to the individual (i.e. blue circumference). From it, if the perimeter increments in 1, the area increments in a magnitude of r+Δr/2. For iso-voxels of 1 mm^3^, if in only 1 slice of the reference is 1 voxel wider than the individual’s brain (Δr=1), a lesion of 2x2 voxels (i.e. 4 mm^2^) could measure 5.628 mm^2^ after affine registration.

**2.3. Evaluation on a sample of 189 stroke brain imaging datasets**

We delineated the index stroke lesion as per the protocol described in the main manuscript (Table 1 on main manuscript) on brain MRI datasets from 187 patients who presented with acute lacunar stroke symptoms and had a recent small subcortical infarct confirmed on MR diffusion imaging (Valdés Hernández et al., 2015). Then, we mapped the delineated lesions on a common “standard” space, and measured the lesion volume before and after the space transformation. Figure S2_4 shows the results of the volumetric measurements on each space. Observe that the increase is not uniform. Statistically, the mean and median of the distribution of the ratio between both measurements slightly differed: the mean increase was 2.6 times increase whilst the median was 2.5 times. This was despite all lesions being localised on the same region (see Valdés Hernández et al., 2015 for lesion distribution analysis).


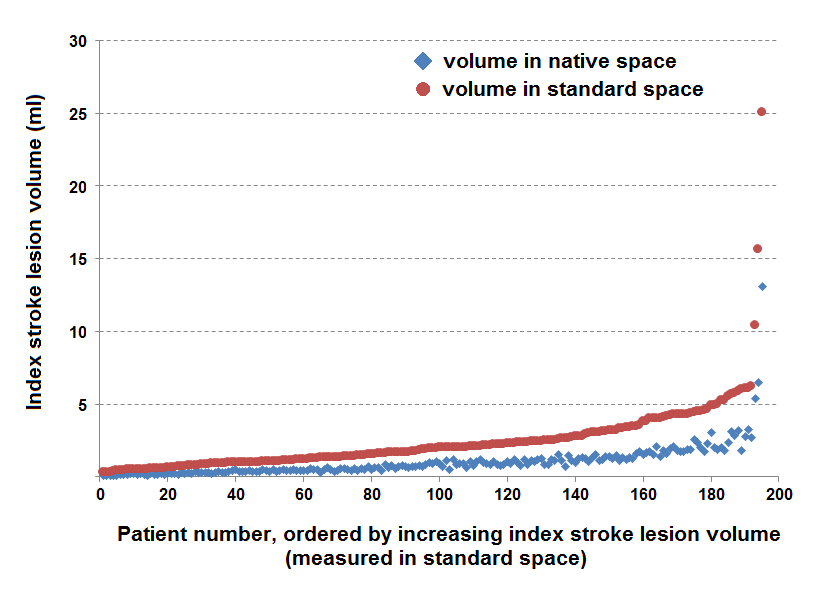


Figure S2_4. Plot of the index stroke lesion volumes of 187 patients with mild stroke, calculated before and after affine aligning all individuals’ brains to a standard brain template.

**References**

Maintz JBA, Viergever MA. A survey of medical image registration. *Med Imag Anal* 1998, 2(1):1-36.

Klein A, Andersson J, Ardekani BA, et.al. Evaluation of 14 nonlinear deformation algorithms applied to human brain MRI registration. *Neuroimage* 2009, 46(3):786-802.

Valdés Hernández MC, Maconick LC, Munoz Maniega S, Wang X, Wiseman S, Armitage PA, Doubal FN, Makin S, Sudlow CL, Dennis MS, Deary IJ, Bastin M, Wardlaw JM. A comparison of location of acute symptomatic versus 'silent' small vessel lesions. *Int J Stroke* 2015; in press.
